# Who can pass the urban filter? A multi-taxon approach to disentangle pollinator trait–environmental relationships

**DOI:** 10.1007/s00442-022-05174-z

**Published:** 2022-05-04

**Authors:** Anika Kristin Gathof, Anita Judit Grossmann, Johann Herrmann, Sascha Buchholz

**Affiliations:** 1 Department of Ecology, TU Berlin, 12165 Berlin, Germany; 2 Institute of Landscape Ecology, University of Münster, 48149 Münster, Germany; 3 Berlin-Brandenburg Institute of Advanced Biodiversity Research (BBIB), 14195 Berlin, Germany

**Keywords:** Connectivity, Functional trait, Hoverflies, Urbanisation, Wild bees

## Abstract

Cities are considered important refuges for insect pollinators. This has been shown repeatedly for wild bees, but may also be true for other diverse taxa such as hoverflies. However, our understanding of how urban environmental filters shape pollinator species communities and their traits is still limited. Here, we used wild bee and hoverfly species, communities and their functional traits to illustrate how environmental filters on the landscape and local scale shape urban species pools. The multi-taxon approach revealed that environmental filtering predominantly occurred at the landscape scale as urbanisation and 3D connectivity significantly structured the taxonomic and functional composition of wild bee (sociality, nesting, diet, body size) and hoverfly (larval food type, migratory status) communities. We identified urban winners and losers attributed to taxon-specific responses to urban filters. Our results suggest that insect pollinator conservation needs to take place primarily at the landscape level while considering species traits, especially by increasing habitat connectivity.

**Supplementary Information:**

The online version contains supplementary material available at 10.1007/s00442-022-05174-z.

## Introduction

Numerous studies in recent years have shown a dramatic decline in insect pollinators (Ollerton et al. 2014; Sanchez-Bayo and Wyckhuys 2019). Due to their multifaceted impacts, the research effort of pollinators has increasingly gained weight (Hall et al. 2017; Baldock et al. 2019), even in the context of functional approaches for a deeper understanding of the underlying mechanisms of environmental filtering in the shaping of species pools (Scheiner et al. 2017). Trait-based ecological knowledge is of great importance for the protection of diverse pollinator populations and, consequently, the preservation of their manifold ecosystem services (Buchholz and Egerer 2020): wild pollinator species are essential to nearly all terrestrial ecosystems (Kevan 1999) as 80% of all flowering plants are pollinated by animals (Ollerton et al. 2011), with bees being the most important group for animal-mediated pollination services globally (Klein et al. 2007; Bates et al. 2011; Ollerton et al. 2011). However, hoverflies are also effective pollinators, contributing to a diversification of pollinator communities with specific characteristics such as pest control and recycling of organic matter within the larval phase as well as long-distance pollen transfer as an adult (Doyle et al. 2020).

One of the most important ways in which environmental changes affect pollinators is through land use change at the regional level. Habitat destruction, degradation and fragmentation caused by agricultural and urban land use drive pollinator population decline (De Palma et al. 2015; Martins et al. 2017). In the face of accelerated urbanisation worldwide and native biodiversity decline, urbanisation is seen as one of the main threats to biodiversity (Ascensão et al. 2018; Baldock et al. 2019) and is often associated with the loss of functional diversity concerning many taxa (Pauw and Louw 2012; Normandin et al. 2017). On the other hand, compared to agro-ecosystems and even nature reserves, some cities can host a greater abundance and diversity of pollinator species due to their complex mosaic structure of different land uses and habitats (Hall et al. 2017; Baldock et al. 2019). Cities are often characterised by a high number of exotic plants, a low pesticide use and a high proportion of sealed surfaces in the form of streets and buildings, which constitute barriers that fragment the urban landscape (Buchholz et al. 2020; Persson et al. 2020). Overall, it is above all the spatial configuration of cities—essentially connectivity or habitat fragmentation—that is a major factor influencing biodiversity and, unlike in agricultural landscapes, connectivity in cities can be multivariate (von der Lippe et al. 2020). There are still significant gaps in our knowledge due to the use of traditional connectivity measures, which did not reflect the 3D mobility of flying insect pollinators (Wenzel et al. 2020).

Urban species pools are determined through environmental filters associated with land use types distributed across the matrix and corresponding connectivity (Aronson et al. 2016). Pollinators are mobile ecosystem service-providing animals with highly diverse life history strategies and functional traits (Kremen et al. 2007), representing a model system to understand the impacts of local to landscape-scale environmental filtering on species pools in an urban landscape matrix. A growing body of research is, therefore, using pollinator communities as a system to investigate the impacts of environmental filters on community assembly processes, often relating pollinator community metrics (species abundance, richness) to environmental factors including impervious surface (Geslin et al. 2016), habitat isolation (Fischer et al. 2016) and fragmentation (Theodorou et al. 2020), as well as habitat management (Blackmore and Goulson 2014) and host plant cover (Banaszak-Cibicka et al. 2016). Yet, existing studies have produced mixed results. For example, bee species richness and abundance either increase or decrease depending on the intensity and the spatial scale of urbanisation (Pereira-Peixoto et al. 2014; Egerer et al. 2017), most likely because most studies focus on traditional biodiversity metrics rather than species traits to explain community assembly processes. Although some previous studies have suggested that responses to urbanisation are highly trait-specific (Theodorou et al. 2020; Wenzel et al. 2020), there is scant knowledge of how specific pollinator traits relate to environmental filters in urban contexts (Banaszak-Cibicka and Zmihorski 2012; Martins et al. 2017; Normandin et al. 2017; Banaszak-Cibicka et al. 2018a; Harrison et al. 2018). Previous studies have obtained different results, making it difficult to derive generalisable trends (Buchholz and Egerer 2020). Yet, some functional wild bee traits have been identified that relate to urbanisation, such as body size and nesting type, whereas other traits, such as diet, do not allow meaningful conclusions to be drawn (Buchholz and Egerer 2020).

The performance of species, which determines their survival, growth and reproduction in their environment, is affected by their functional traits (Kearney et al. 2021). Due to distinctive trait expressions, organisms respond differently to biotic and abiotic conditions both inter- and intraspecifically. Predicting how species respond to unique urban conditions requires an understanding of how functional traits respond to their environmental conditions. Certain traits can determine whether a species is able to cope with the prevailing environmental conditions and consequently emerges as a winner or loser (Banaszak-Cibicka and Zmihorski 2012). Indicator species can be used as estimators for environmental conditions (Caro and Doherty 1999) and allow conclusions to be drawn about which trait combinations are suitable to pass the urban filter. Since trait expression is species-specific, a wide range of species, including different taxa, needs to be studied to assess the overall impact of cities on pollinators.

Specific abiotic environmental factors and the mosaic of land use patterns across an urban landscape matrix can support diverse pollinator communities by providing manifold ecological niches for various species with different habitat requirements and functional traits (Banaszak-Cibicka and Żmihorski 2012). However, not all pollinators will be able to claim urban habitats in the same way due to different functional traits. This is the case, for example, with wild bees and hoverflies, which may have different habitat requirements due to differences in their biology and ecology, especially regarding the larval development and the use of floral resources. Hoverflies are considered pollinator generalists, whereas many wild bee species have a close relationship to specific plants (oligolectic diet) (Perrson et al. 2020; Doyle et al. 2020; Westrich 2019). Therefore, multi-species approaches are essential to understand the biodiversity dynamics of urban pollinator communities, also at the functional level.

Most studies examining the functional diversity of pollinators in urban environments rely on descriptive methods. Only a few utilise statistics on functional diversity indices and trait–environment relationships to analyse how traits vary across environments (Buchholz and Egerer 2020). In the context of pollination, there is less information on the effects of urbanisation on non-bee-taxa in general and hoverflies in particular (Senapathi et al. 2017), although hoverflies are proven to be important pollinators of wild plants (Ollerton et al. 2011; Persson et al. 2020) and crops (Klein et al. 2007). The few studies that exist documented a negative impact on hoverflies and a decline in abundance and diversity along rural-to-urban gradients (Bates et al. 2011; Verboven et al. 2014; Baldock et al. 2015; Persson et al. 2020), while functional traits have not yet been studied in detail.

We examine how local and landscape-level filters shape wild bee and hoverfly communities and traits in urban environments. We aim to identify which local (patch size, cover of herbaceous and non-native plants, mean air temperature) and landscape (urbanisation, 3D connectivity) factors are most predictive of the communities and traits found in cities. Specifically, we aim to answer the following questions: (1) which urban landscape matrix factors and local habitat factors affect the composition of wild bee and hoverfly communities? (2) can species be identified that occur exclusively or more frequently in urban or respectively rural areas, so-called “indicator species”? (3) which urban matrix and local habitat factors relate to the functional traits of wild bee and hoverfly communities?

## Materials and methods

### Study system and area

Our study was performed in the administrative region of Berlin, spanning 891.1 km^2^ and inhabiting a population of approximately 3.6 million people. We used the CityScapeLab Berlin (von der Lippe et al. 2020) as our study system, a novel research platform that allowed us to study urbanisation effects on biodiversity patterns of pollinators and uses urban grassland as a model ecosystem; urban grassland is an essential component of urban green spaces (Fischer et al. 2013; Klaus 2013) and a potentially important habitat especially for wild bees (Hall et al. 2017; Dylewski et al. 2019) and for the foraging of adult hoverflies. We, therefore, studied 49 urban grasslands distributed across the city, 44 located in and 5 outside of Berlin. Each study site consisted of a dry grassland patch that encompasses one randomly located plot with a standardised size (4 × 4 m) for sampling environmental variables (vegetation variables and temperature) and pollinators (von der Lippe et al. 2020).

### Pollinator sampling

We sampled pollinators in summer 2017, using pan traps across three sampling rounds approximately 6 weeks apart (29 May to 02 June, 03 July to 07 July, 04 Sep to 08 Sep). In each sampling round, the traps were set up for 72 h and emptied afterwards. Pan trapping is a common passive method for catching pollinators based on visual attraction (Kearns and Inouye 1993; Dafni et al. 2005) and has also been used in other studies that focussed on both wild bees and hoverflies (Bates et al. 2011; Persson et al. 2020). This approach was used because it allowed us to simultaneously sample all 49 sites using the same sampling effort, reduce collector bias and temporal bias, obtain a standard estimate of pollinator species richness and abundance co-occurring within a site (Westphal et al. 2008; Devigne and De Biseau 2014). Pan traps bias bee collection towards small-bodied bees (Cane et al. 2000). We assumed that the systematic bias introduced by the collection of samples with pan traps would be consistent across all our study plots.

After colouring the plastic bowls (radius 7.25 cm, depth 5 cm) by spraying them yellow, blue and white with Sparvar Leuchtfarbe (Spray-Color GmbH, Merzenich, Germany), we placed a triplet of pan traps on the study sites. We used three different colours because studies have shown that this increases the catching performance by attracting more pollinator species (Vrdoljak and Samways 2012). We pinned the plastic bowls to two wood sticks at vegetation height (approximately 30 cm above the ground) and filled them with approximately 300 ml of 4% formaldehyde solution and one drop of detergent to break the surface tension. Good weather conditions for pollinator activity were taken into account when selecting sampling sessions (minimum of 15 °C, low wind, no rain and dry vegetation). All caught pollinators were dried, pinned and identified to species level using standardised identification keys for bees (Amiet 1996; Amiet et al. 1999, 2001, 2004, 2007, 2010; Gokcezade et al. 2010) and hoverflies (Bartsch et al. 2009a, b). Since we focussed only on wild pollinators in this study, we excluded the 510 caught specimens of *Apis mellifera* (the honeybee) from the data set since their abundance follows seasonal patterns other than those of wild bees (Tommasi et al. 2004). The taxonomy of wild bees followed the nomenclature of Scheuchel and Willner (2016) and that of hoverflies the nomenclature of Speight et al. (2013).

### Pollinator traits

To investigate the trait–environmental relationships of wild bee and hoverfly communities, we derived functional traits for each pollinator species from literature (Table 1). For wild bees, we used [WB 1] sociality; [WB 2] nesting behaviour; [WB 3] diet; [WB 4] active flight time and [WB 5] body size from the anterior extremity of the head (excluding the antennae) to the posterior extremity of the abdomen (Amiet 1996; Amiet et al. 1999, 2001, 2004, 2007, 2010; Westrich 2019). For hoverflies, we derived [HF 1] body size of adult individuals from head to the end of the abdomen, [HF 2] active flight time, [HF 3] migratory status (Speight et al. 2013) and [HF 4] larval food type (Saure 2018).

**Table 1:** Selected functional traits of pollinator species

Functional traits	Unit/score/categories	Explanation
Wild bees		
[WB 1] Sociality	Solitary	Nest foundation occurs alone; no division of labour and no storage of food stocks
	Eusocial	Organised society, which includes division of labour between the nest founder (queen) and the workers; short-lived (usually one growing season). All levels of social life have been included
	Cleptoparasitic	Brood parasitic way of life
[WB 2] Nesting behaviour	Hypergeic	Aboveground nesting; cavities in trees and masonry, plant stems, snail shells and between rocks
	Endogeic	Belowground nesting in self-excavated or existing cavities
	Hyper- and endogeic	Combination of the two nesting types
	Cleptoparasitic	Penetration into foreign nests and deposition of the eggs therein
[WB 3] Diet	Oligolectic	Pollen specialisation
	Polylectic	No binding to certain plant species
	Cleptoparasitic	No collection of pollen
[WB 4] Active flight time	# Average number of months	Average flight period of female and male individuals
[WB 5] body size	# Average size in mm	Average size of female individuals from the anterior extremity of the head (excluding the antennae) to the posterior extremity of the abdomen
Hoverflies		
[HF 1] body size	# Average size in mm	Average size of adult individuals from the anterior extremity of the head (excluding the antennae) to the posterior extremity of the abdomen
[HF 2] active flight time	# Average number of months	Average flight period of female and male individuals
[HF 3] migratory status	0/1 (no/yes)	Ability to undertake long-distance movements
[HF 4] larval food type	Phytophagous	Nutrition through plant components
	Zoophagous	Nutrition through animals, especially aphids
	Terrestrial saprophagous	Nutrition through microorganisms in terrestrial substrates (rotting herbaceous plants, dung, tree hollows, sap flows, etc.)
	Aquatic saprophagous	Nutrition through microorganisms in aquatic substrates (puddles, wastewater, liquid manure or mud at the bottom of waters)

### Environmental variables

We determined 6 environmental variables at 2 spatial scales to describe the setting of 49 study sites and could fall back on the preliminary analyses of the CityscapeLab Berlin (von der Lippe et al. 2020). For describing the urban matrix, we used the variables [1] urbanisation and [2] 3D connectivity (Table 2) (von der Lippe et al. 2020). Both variables have been identified as important predictors for wild bee community composition within cities in previous studies (Geslin et al. 2016; Martins et al. 2017), although for connectivity, only two dimensions have been included so far. For [1], we relied on a frequently used urbanisation measure and used the proportion of impervious surface (Fortel et al. 2014; Geslin et al. 2016; Choate et al. 2018) within a 500-m buffer around the plot (SenUDH 2011). We chose a buffer radius of 500 m because this distance reflects the radius of action of most wild bees (Zurbuchen et al. 2010). Although hoverflies can be more mobile (Lysenkov 2009; Doyle et al. 2020), they are also sufficiently considered in this buffer radius. As pollinators are mobile and use airspace in particular, our [2] 3D connectivity variable is based on Hanski’s habitat connectivity index (Hanski 1994, 1999) and combines area sizes with distances to other dry grasslands (SenUDH 2014a) and building heights to provide a 3D connectivity. The factor weighting the distance that originally describes the dispersal capacity of species was modified to take into account the 3D urban landscape context. To do so, building heights (SenUDH 2014b) in corridors of 25 m around the connecting lines between patches were summed up and added to the distance calculation. The distance thus increases with more and higher buildings in between patches (resulting in less connectivity). Spatial analyses for urbanisation was performed using QGIS Version 2.18.11, applying the tools Edge distance vector of the Conefor Inputs plugin (Saura and Torné 2009) and Zonal statistics and ArcGIS 10.3.1, using the tool Generate Near Table for 3D connectivity.

**Table 2:** Environmental variables of the CityScapeLab Berlin (von der Lippe et al. 2020) used for statistical analyses of predictors

Variable	Unit	Explanation	Method/equipment/software	Data source/reference
Urban matrix				
[1] Urbanisation	%	Percentage of impervious surface in a 500-m buffer around the biotope patch in which the plot is located	Software: QGIS version 2.18.0; tool: Zonal statistics	Berlin Environmental Atlas/Actual Use of Built-up Areas, Inventory of Green and Open Spaces 2010 (SenUDH 2011)
[2] 3D connectivity	0-…/#	Hanski’s connectivity index (modified): considers area size, distance to other dry grassland patches and buildings height	Software: ArcGIS 10.3.1 for Desktop; tool: Generate Near Table (Analysis)	Berlin Environmental Atlas/Biotope Types (SenUDH 2014a), Biotope mapping Brandenburg (Landesamt für Umwelt Brandenburg 2009); Berlin Environmental Atlas/Building and Vegetation Heights (SenUDH 2014b) Hanski (1994, 1999)
Local habitat scale				
[3] Patch size	m^2^	Size of the dry grassland patch in which the plot is located	Software: QGIS version 2.18.0	Berlin Environmental Atlas/Biotope Types (SenUDH 2014a), Biotope mapping Brandenburg (Landesamt für Umwelt Brandenburg 2009)
[4] Cover of herbaceous plant species	%	Estimated cover of herbaceous plants on the plot (no grasses, no coniferous trees) herbaceous plants potentially form flowers and thus represent the main resource source for many pollinators	Estimation flowering ability derived from Biolflor (Klotz et al. 2002)	
[5] Cover of non-native herbaceous plant species	%	Estimated cover of non-native herbaceous plants on the plot (no grasses, no coniferous trees)	Estimation floristic status derived from Biolflor (Klotz et al. 2002)	
[6] Mean air temperature	°C	Mean maximum air temperature of the plot measured at 2 m between April and September (month selection was based on the pollinator activity periods)	Data logger: EasyLog EL-USB-2 + , Lascar Electronics	

At the local scale, we used the variables [3] patch size, [4] cover of herbaceous plants, [5] cover of non-native herbaceous plants and [6] mean air temperature (Table 2) to characterise the local habitat features of each grassland patch. The cover of herbaceous plants was collected within each plot (4 × 4 m) as a measure of local resource availability, using the Braun-Blanquet approach, which lists the plant species present in order of layers (trees, shrubs, herbaceous plants) and scores them according to the degree of cover (van der Maarel and Franklin 2012). Further, we considered the cover of non-native herbaceous plants because previous studies have shown that non-native plants possibly cause novel ecosystem interactions (Schweiger et al. 2010; Schirmel et al. 2016; Davis et al. 2018). For this variable, the coverage of all non-natives was summed up. To describe the urban-influenced microclimate of each plot, we summarised the air temperature values measured every 10 min from April to September 2017 (EasyLog EL-USB-2+, Lascar Electronics) and averaged them per plot to obtain the variable mean air temperature.

### Data preparation and statistical analysis

To determine (1) how the species composition of wild bees and hoverflies relates to our set of environmental variables, we used non-metric multidimensional scaling (NMDS). For this purpose, species found only once or twice and sites with less than three individuals were omitted from the data set beforehand to enhance the accuracy of statistical analyses and to reduce statistical noise. Thus, 51 bee species with 875 individuals from 46 sites and 16 hoverfly species with 1228 individuals from 47 sites were each used in the NMDS. The relative abundances of these species were standardised by performing Wisconsin double standardisation and square-root transformation. The Bray–Curtis dissimilarity matrix of each pollinator group was used for scaling, and a maximum number of 100 random starts was conducted in search of a stable solution. The environmental variables were fit onto the ordination with 99,999 permutations to assess the significance of correlations between species and these factors.

For (2) identifying indicating pollinator species, we calculated indicator values (IndVal) for the significant variables assessed in the NMDS. For urbanisation, the following levels were defined: low (≤ 20% of impervious surface in a 500-m buffer around the biotope patch in which the plot was located), medium (> 20– ≤ 50%) and high (> 50%). The 3D connectivity was divided into the following levels: low (≤ 0.03 values of Hanski’s connectivity index), medium (> 0.03 ≤ 0.10) and high (> 0.10). Indicator species, characterised by specificity (abundant in a specific type of habitat) and by fidelity (predominantly found in this type of habitat), were calculated as the product of the relative frequency and relative average abundance in cluster with the package labdsv in R (Dufrêne and Legendre 1997; Roberts 2016). Pollinator species with indicator values of ≥ 0.3 were defined as indicator species.

For research question (iii), we used a combined RLQ method and fourth-corner analysis with the “ade4” package v. 1.7–11 (Dray and Dufour 2007) to determine how the urban matrix and local habitat factors may filter pollinator traits in urban grasslands. The RLQ method was used to summarise the joint structural relationships (in the data sets) between the environmental filters, wild bee and hoverfly abundances, and each trait distribution among grasslands. We then used the fourth-corner analysis to test for correlations between environmental filters and pollinator traits (Dray and Legendre 2008; Dray et al. 2014). Wild bees and hoverflies were analysed separately. This involved creating three matrices for each pollinator group: an R matrix (environmental variables), an L matrix (wild bee/hoverfly species abundances) and a Q matrix (wild bee/hoverfly species traits). Subsequently, a correspondence analysis (L matrix) and principal components analysis (R, Q matrices) were performed while applying a permutation model using model type 2, that permutes the values of the sites (rows of L matrix). Further regressions, using Poisson GLMs, were applied to determine the direction of the effect. Therefore, we counted the frequency of each trait per species community or study site, respectively, and used these count data as response variable to environmental variables. All analyses were performed in the R Statistical Environment (version 3.3.1, R Core Team 2016), including the packages ade4 (Dray and Dufour 2007), labdsv (Roberts 2016), AER (Kleiber and Zeileis 2008) and vegan (Oksanen et al. 2013).

## Results

In total, 953 specimens of wild bees (Apoidea excluding *A. mellifera*) from 106 species were collected and 1.246 hoverflies (Syrphidae) from 31 species (Appendix 1). The most abundant wild bee species were *Lasioglossum morio* (139 individuals), *Lasioglossum calceatum* (95 individuals), *Bombus terrestris* (67 individuals) and *Lasioglossum laticeps* (51 individuals). We found 26 Red-Listed (Berlin) wild bee species (Saure 2005). The most abundant hoverfly species were *Helophilus trivittatus* (610 individuals), *Helophilus pendulus* (245 individuals), *Eristalis arbustorum* (192 individuals), and *Episyrphus balteatus* (62 individuals). We identified two Red-Listed (Berlin) hoverfly species (Saure 2018).

**Fig. 1:**
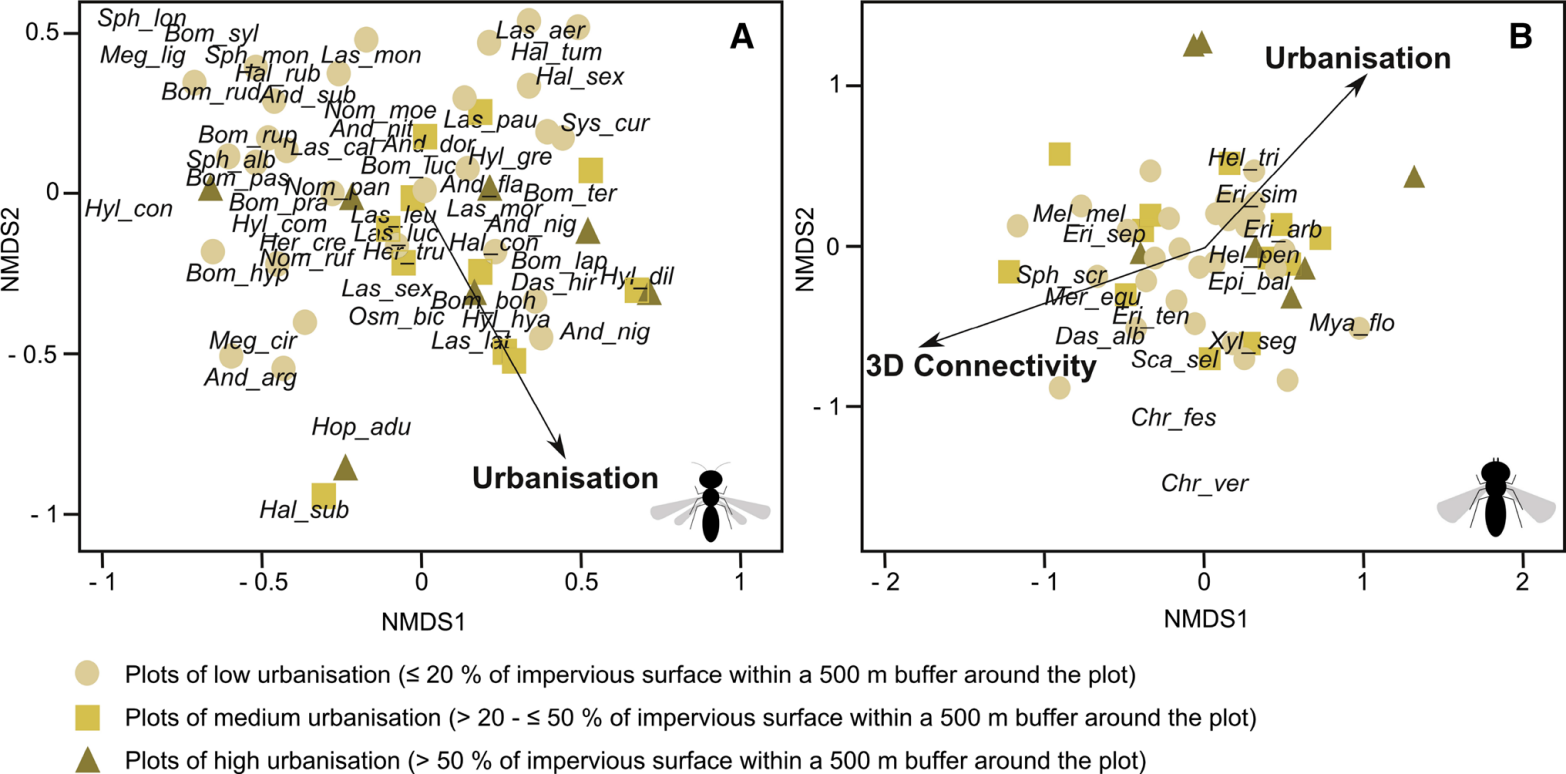
Non-metric multidimensional scaling (NMDS) plots showing **A** wild bee and **B** hoverfly community composition in relation to landscape-scale features (urbanisation and connectivity) and local features. Sites are classified according to their degree of urbanisation (circles: low urbanisation; squares: medium urbanisation; triangles: high urbanisation). The significant variables (*p* < 0.05) are shown with an arrow

The species composition of wild bees was significantly affected by urbanisation (*P* = 0.043*). The NMDS plot (Fig. 1) shows a slight grouping of study sites, indicating differences in the distribution of wild bees among three levels of urbanisation. Bee species showed different responses to urbanisation. Certain species, e.g. *Dasypoda hirtipes*, *L. morio*, seemed to cope well in highly urban surroundings, whereas others (*Bombus rupestris*, *Lasioglossum pauxillum*) showed the opposite pattern. The species composition of hoverflies was significantly affected by urbanisation (*P* = 0.0064**). The NMDS plot shows that the majority of species (e.g. *Chrysotoxum verralli, Dasysyrphus albostriatus*) is not aligned to urbanisation, while a few species (e.g. *Eristalis similis, Helophilus trivittatus*) appear to be more adapted due to their proximity to the urbanisation vector. Furthermore, 3D connectivity had a significant effect on hoverfly species composition (*P* = 0.0031**). Both 3D connectivity and urbanisation structured hoverfly communities but in opposing ways.

**Fig. 2:**
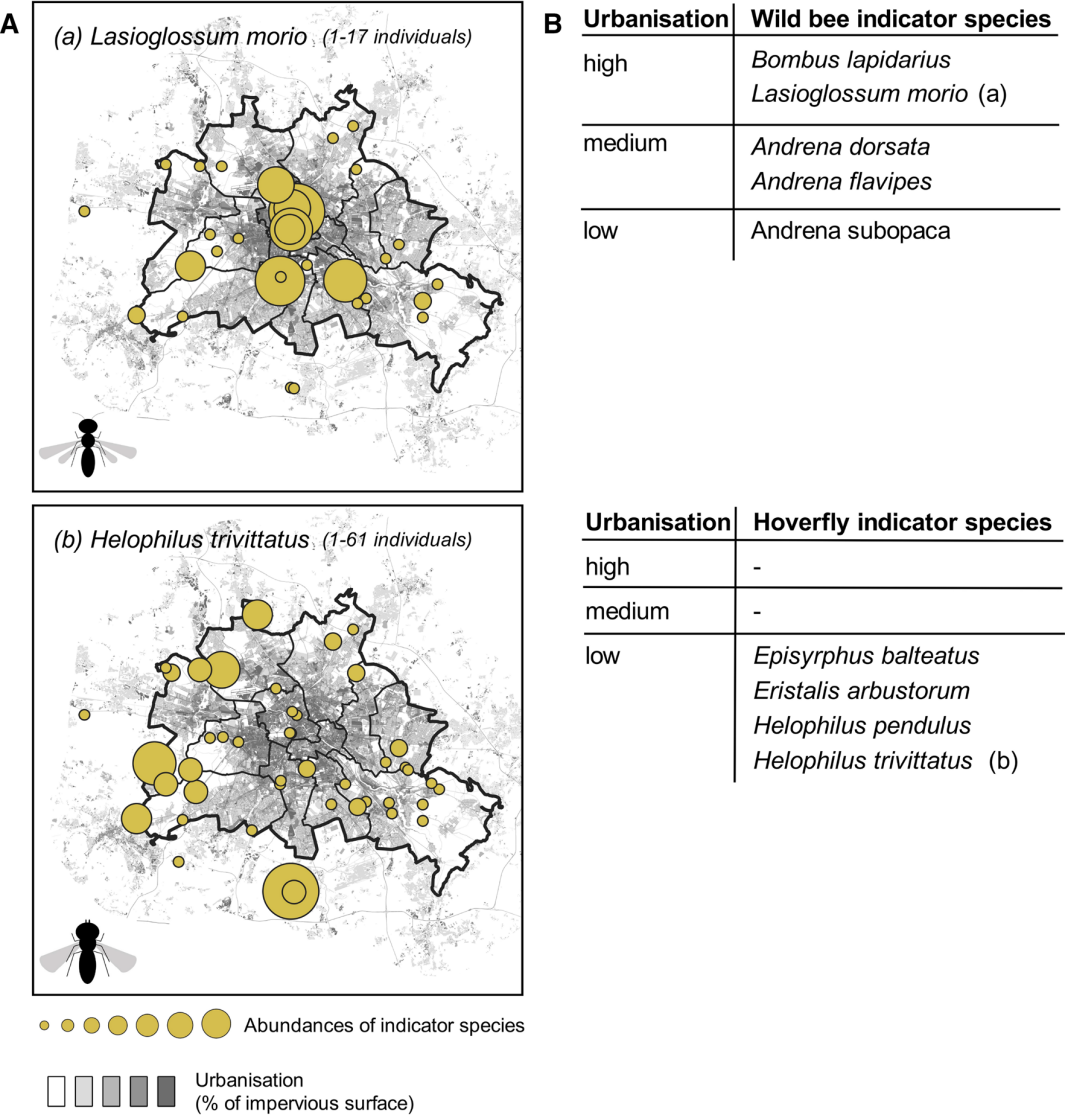
**A** Abundances of the indicator species *L. morio* (high urbanisation) and *Helophilus trivittatus* (low urbanisation) in the study sites across the urban matrix of Berlin. **B** List of indicator species for the three levels of urbanisation, namely low (≤ 20% of impervious surface in a 500 m buffer around the biotope patch in which the plot is located), medium (> 20– ≤ 50%) and high (> 50%)

Indicator value analysis revealed indicator species for wild bees and hoverflies for different levels of urbanisation and 3D connectivity. *L. morio* (IV = 0.37) and *Bombus lapidarius* (IV = 0.32) were the only species identified as indicators for high levels of urbanisation as they were predominantly recorded at highly urbanised sites (Fig. 2). The bee species *Andrena flavipes* (IV = 0.36) and *Andrena dorsata* (IV = 0.32) were related to medium urbanisation and *Andrena subopaca* (IV = 0.39) to low urbanisation. Regarding hoverflies, *Helophilus trivittatus* (IV = 0.5492) (Fig. 2), *Episyrphus balteatus* (IV = 0.5546), *Eristalis arbustorum* (IV = 0.4207) and *Helophilus pendulus* (IV = 0.4790) were identified as indicator species for low levels of urbanisation. The Indicator Value Analysis further identified *Helophilus trivittatus* (IV = 0.514) and *Helophilus pendulus* (IV = 0.402) as indicators for medium 3D connectivity, whereas *Eristalis arbustorum* (IV = 0.3944) indicated low 3D connectivity.

Regarding wild bees, RLQ analysis revealed a highly significant positive relationship between urbanisation and polylectic species (*P* = 0.0052**) and a significant positive relationship was also found for eusocial species (*P* = 0.0401*) and endogeic species that nest exclusively belowground (*P* = 0.0495*) (Table 3). Further, urbanisation favoured small-sized bee species (*P* = 0.0353*). Cleptoparasitic species in turn responded negatively to urbanisation in terms of diet (*P* = 0.0010**), nesting behaviour (*P* = 0.0010**) and sociality (*P* = 0.0016**). Regarding hoverflies, urbanisation influenced the larval food type as it increased the number of species with a terrestrial saprophagous larval phase (*P* = 0.0390*) (Table 3). Furthermore, it had a significant effect on the migratory status as it negatively impacted the abundance of migrating hoverflies (*P* = 0.000401***). The 3D connectivity only affected functional groups of wild bees but not of hoverflies. Contrary to urbanisation, 3D connectivity decreased the abundance of eusocial bee species (*P* = 0.0018**) and favoured large-sized bee species (*P* = 0.0018*). At the local scale, no significant relations between habitat variables and functional traits were found.

**Table 3:** Trait–environment relationships in urban wild bee and hoverfly assemblages (fourth-corner analysis)

Taxon	Functional trait		Urbanisation	Connectivity
Wild bees	Sociality	Eusocial	+*	-**
		Cleptoparasitic	-**	
	Nesting	Endogeic	+*	
		Cleptoparasitic	-**	
	Diet	Polylectic	+**	
		Cleptoparasitic	-**	
	Body size		-*	+*
Hoverflies	Larval food type	Terrestrial saprophagous	+*	
	Migratory status	Migrating	-***	

Asterisks show positive (+) or negative (-) trait–environment relationships with *P* < 0.05. All relationships with 0.01 < *P* < 0.05 are shown with one asterisk (*), relationships with 0.001 < *P* < 0.01 are depicted with two asterisks (**), and relationships with *P* < 0.0001 are shown with three asterisks (***)

## Discussion

Our multi-taxon approach revealed that environmental filtering predominantly occurred at the landscape scale as urbanisation and 3D connectivity significantly affected the taxonomic and functional composition of wild bee and hoverfly communities whereas habitat conditions, such as the availability of plant resources, did not play a significant role in our study. Wild bee species may respond positively to urbanisation as these species likely benefit from urban features such as higher temperatures, the high diversity and year-round availability of floral resources and various nesting opportunities within the urban matrix (Baldock et al. 2019; Zaninotto et al. 2020). For example, *L. morio*—an indicator species for high levels of urbanisation in our study—is ubiquitous as it colonises various habitats (Westrich 2019) and was predominantly found in urban areas. It is a good example for a typical ‘urban winner’ that prefers xero-thermophilic conditions (Geslin et al. 2015; Passaseo et al. 2020), is adaptive in its choice of nesting substrate and uses a wide range of pollen resources (Westrich 2019). In addition, *Andrena dorsata* and *Andrena flavipes*, with similar ecological requirements, seemed to tolerate a certain amount of urbanisation, which can be explained by their ability to colonise various habitats, also in residential areas, as well as their polylectic diet (Westrich 2019). In contrast, hoverflies seem to be more sensitive to urbanisation and perform better in low-urbanised habitats, which is also evidenced by Verboven et al. (2014), Baldock et al. (2015) and Persson et al. (2020). Due to their demands in the larval phase, many hoverfly species have a strong biotope attachment and prefer more humid, wooded and rather cooler habitats than dry grasslands (Saure 2018). However, as pollinator generalists and highly mobile flying insects in their adult phase, hoverflies are able to exploit a wide range of nectar- and pollen-plants and thus probably benefit from well-connected habitat structures to access spatially widespread resources. Enhancing 3D connectivity, meaning shorter distances between patches and lower building heights, may mitigate negative effects of urbanisation on hoverflies, which cover long flight distances (Doyle et al. 2020). Based on their contribution to pollination and pest control, the conservation of hoverflies is important to maintain ecosystem services in urban areas. Contrary, 3D connectivity had no effect on wild bee assemblage presumably due to the suitability of the study system dry grassland as habitat for bees (Saure 2005) and their limited home range. Certain bee species, especially small-bodied bees have a very small activity radius, at times as small as 100 m (Westrich 2019), which allows them to use sites in close proximity only. Moreover, many wild bee species are specialised on certain plants but might be less dependent on connectivity if all required floral and nesting resources are available within their home range. These results indicate that the influence of connectivity as a predictor for biodiversity is context-dependent and habitat suitability can play a critical role in shaping urban pollinator communities. Thus, 3D connectivity in particular should be implemented in future pollinator research.

Several studies have shown a significant effect of urbanisation on wild bee assemblages and functional traits (Banaszak-Cibicka and Żmihorski 2012, 2020; Verboven et al. 2014; Fischer et al. 2016; Martins et al. 2017) and on hoverfly assemblages (Bates et al. 2011; Verboven et al. 2014; Baldock et al. 2015; Persson et al. 2020). However, most studies examining the relationships between pollinator traits and urbanisation were not able to identify generalisable trends (Buchholz and Egerer 2020). This is mostly due to the fact that only a limited number of studies (Braaker et al. 2017; Harrison et al. 2018; Buchholz et al. 2020) used a multifunctional approach by applying appropriate statistics such as RLQ- and fourth-corner analyses (Buchholz and Egerer 2020). Based on these statistics, our study highlights that urbanisation shaped the assemblages of both bees and hoverflies by selecting a specific set of traits. Urban dry grasslands can act as refuges, for example for endogeic [belowground nesting] bees, as they offer nesting opportunities in the form of bare soil even within highly urbanised surroundings. This is in contrast to studies that found urban areas to benefit hypergeic [aboveground nesting] species (Banaszak-Cibicka and Żmihorski 2012; Bates et al. 2011; Neame et al. 2013; Fortel et al. 2014), but Theodorou et al. (2020) also highlighted the potential of sparsely managed fragments of semi-natural vegetation—such as dry grasslands—for endogeic species. The increase in eusocial bees in the context of urbanisation may be explained by the ecological dominance of social insects due to greater adaptability to unfavourable conditions compared to solitary species (Chapman and Bourke 2001). Resilience is based on the community structure, the ability to use resources efficiently and store food, large numbers of individuals, and collective defence against disturbances (Westrich 2019). In addition, dry grasslands offer diverse nesting substrate for colony-building species (Cane et al. 2006), which often nest belowground.

Our findings also indicate a decrease in cleptoparasites with increasing urbanisation, similar to the results of other studies that recorded comparatively less cleptoparasitic bee species in urban habitats (Lerman and Milam 2016; Banaszak-Cibicka et al. 2018a). To persist, parasitoid insects depend on the availability (Corcos et al. 2019) and large populations of their host species (Matteson et al. 2008). Parasites in general can impact local biodiversity, including the functional level, by reducing host abundance and correspondingly increasing trait diversity by regulating the occurrence of dominant species (Frainer et al. 2018). However, cleptoparasites made up only a small proportion (5%) of all recorded bees, and thus, related results must not be overstated. The positive response of polylectic bee species to urbanisation is in line with the findings of several studies investigating diet-related traits for urban and rural landscapes (Matteson et al. 2008; Ahrné et al. 2009; Antonini et al. 2013; Deguines et al. 2016). Polylectic bee species can benefit from a wide variety of flowering plants, including ornamentals, which are abundant in urban areas due to the diversity of parks, gardens and other green spaces (Eremeeva and Sushchev 2005). Surprisingly, oligolectic bees were not disadvantaged by urbanisation, in contrast to the findings of most studies as shown in a recent review (Buchholz and Egerer 2020), which could be attributed to the suitability of dry grasslands (plant species richness, open soil, low management) even for specialised bee species.

Supporting the findings of previous studies, our analyses revealed that urbanisation favours small-bodied bees (Ahrné et al. 2009; Banaszak-Cibicka and Żmihorski 2012; Wray et al. 2014; Hamblin et al. 2018; Eggenberger et al. 2019), which require lower amounts of nectar and pollen to reproduce than large-sized species (Cane et al. 2006). Thus, they can persist in small habitat patches with limited but sufficient floral resources in a fragmented urban surrounding (Greenleaf et al. 2007; Banaszak-Cibicka et al. 2018b). Contrary to urbanisation, connectivity filters for large-bodied bee species. Connectivity within urban areas may enable bees of large size to access suitable floral resources in the surrounding landscape to cover their greater foraging needs (Wray et al. 2014; Cresswell et al. 2000). Species of large body size that are disadvantaged by urbanisation can thus benefit from well-connected habitats within the urban landscape.

Regarding hoverflies, habitat requirements within the larval phase could explain the performance of this pollinator group in urban areas (Verboven et al. 2014; Persson et al. 2020). As the required local microhabitat structures for terrestrial saprophagous larval phases can be found in the form of rot-holes, tree-hollows, compost heaps and dung in residential gardens, parks, cemeteries and other urban green spaces, species with this larval development type may thrive in urban habitats. In terms of migratory status, our results mirror those of Luder et al. (2018), which is the only study on this research topic and which detected fewer migratory species in urban than in rural sites. Their high mobility probably enables these species to access floral and larval food resources spread across a large area. In general, cities provide high small-scale resource heterogeneity, but relevant resources are often less abundant in urban compared to rural areas.

## Conclusion

Urban matrix variables at the landscape scale, namely urbanisation and 3D connectivity, have a filtering effect on the functional traits of both pollinator groups and thus form specific urban species assemblages. This suggests that biodiversity conservation needs to take place primarily at the landscape level, especially through the provision of stepping-stone habitats, linking habitats by increasing connectivity. Here, not only imperviousness plays a role but also building height, as the effect of 3D connectivity clearly shows. This does not mean that habitat-level measures such as flower strips or extensive management are not important, but they need to be thought of in a wider spatial context. For example, the best flowering strip will not be very promising if it is located in an isolated backyard of a building complex.

Our results show that urban habitats can have a great potential for diverse pollinator communities. Nevertheless, an understanding of the underlying mechanisms how pollinator assemblages are shaped in urban environments is of high relevance for future conservation strategies, but it must be based on robust data analyses with appropriate statistical tools. In this way, it is possible to define winners and losers of increasing urbanisation and to adapt species protection measures in a targeted manner. For example, endogeic species could be promoted by the provision of open sand or soil patches. These are, at the same time, also important stepping-stone habitats. Species that are disadvantaged by urbanisation (large-bodied bees, hoverflies) can be promoted by providing well-connected urban green areas such as flower strips, which can easily be realised in the roadside greenery and not only selectively distributed across the city. For hoverflies, well-connected urban green areas should also include diverse microhabitat structures, especially various water bodies.

Our study highlights the importance of functional approaches. Although there are already several studies that shed light on the interaction of urban matrix variables and life history traits, there is still a lot of work to be done in this growing field. It would be desirable to conduct cross-city and cross-habitat studies to derive more generalisable conclusions on the one hand and to shed more light on the effect of the landscape level and the configuration of the urban matrix on the other. In this way, tailor-made biodiversity protection measures can contribute to the sustainable development of green cities of the future. Cities could thus become sustainable real laboratories for the protection of biodiversity.

## Supplementary Information

Below is the link to the electronic supplementary material.

Supplementary file1 (XLSX 19 KB)

## Data Availability

Our data will be provided as electronic supplementary material.
